# Fine-tuning TrailMap: The utility of transfer learning to improve the performance of deep learning in axon segmentation of light-sheet microscopy images

**DOI:** 10.1371/journal.pone.0293856

**Published:** 2024-03-29

**Authors:** Marjolein Oostrom, Michael A. Muniak, Rogene M. Eichler West, Sarah Akers, Paritosh Pande, Moses Obiri, Wei Wang, Kasey Bowyer, Zhuhao Wu, Lisa M. Bramer, Tianyi Mao, Bobbie Jo M. Webb-Robertson

**Affiliations:** 1 AI & Data Analytics Division, Pacific Northwest National Laboratory, Richland, WA, United States of America; 2 Vollum Institute, Oregon Health & Science University, Portland, OR, United States of America; 3 Biological Sciences Division, Pacific Northwest National Laboratory, Richland, WA, United States of America; 4 Appel Alzheimer’s Disease Research Institute, Feil Family Brain and Mind Research Institute, Weill Cornell Medicine, New York, NY, United States of America; University of Illinois Urbana-Champaign, UNITED STATES

## Abstract

Light-sheet microscopy has made possible the 3D imaging of both fixed and live biological tissue, with samples as large as the entire mouse brain. However, segmentation and quantification of that data remains a time-consuming manual undertaking. Machine learning methods promise the possibility of automating this process. This study seeks to advance the performance of prior models through optimizing transfer learning. We fine-tuned the existing TrailMap model using expert-labeled data from noradrenergic axonal structures in the mouse brain. By changing the cross-entropy weights and using augmentation, we demonstrate a generally improved adjusted F1-score over using the originally trained TrailMap model within our test datasets.

## Introduction

Understanding how functional brain states are achieved and how they are modulated by experience, genetics, and pharmacological agents require a reference map of the structural connections between neurons. Such a map will generate insights into the information processing mechanisms of healthy brains but also facilitate research into brain disorders, which is the goal of the Mouse and Human Connectome Projects [1–3].

Light-sheet fluorescence microscopy (LSFM) is an important tool in this discovery process. Based on wide-field fluorescence technologies, LSFM enables the visualization of cellular structure at a micrometer scale and allows optical sectioning by automating the movement of a sample through a plane of light. The resulting fluorescence is detected by an orthogonally-placed camera. By only illuminating a thin section of tissue with each pass, photobleaching of the tissue is minimized, yielding a more intense and complete fluorescence signal. Another important parallel advancement is optical tissue clearing. Through the use of organic solvents, lipid removal, or immersion in refractive index matching solutions, brain tissue is rendered effectively transparent, allowing greater contrast of the labeled fluorescent structures [4–6].

Combining LSFM and brain clearing enables the tracing and analyzing of whole brain connectivity at a mesoscopic level. However, technical challenges remain to make this high-resolution data meaningful for connectome research, specifically: to stitch together aligned serial sections to construct a 3D image volume; to efficiently annotate voxels of interest (for example, labeling the content as belonging to the axon of a particular neuron); and to register landmarks in an image volume with a reference atlas so that data from multiple studies might be compared in the same coordinate space [7].

Manual annotation is one approach to addressing some of these challenges. However, annotating a volume of images is a laborious and time-intensive process that can be subjective due to fatigue-related errors and inhomogeneities in the intensity of stained structures resulting in significant variability between experimenters. In addition, the number of image slices that yield a complete volume range from several dozen to over ten thousand, depending on the microscopy system, species, and orientation of the sectioning method [8, 9]. A complete dataset of high-resolution images for a whole brain can be on the order of a terabyte, limiting analysis algorithms to those that do not require the full volume of data to be loaded into run-time memory [10].

Computational methods to automatically stitch, segment, annotate, and register meaningful information in images have made enormous strides in the past decade. Early methods tended to rely on thresholding, clustering, edge detection, region growing, or curve propagation [11, 12]. Algorithms have also been developed to trace axons, such as the all-path-pruning algorithm, which uses a starting seed-location, usually of the soma of the neuron, to trace 3D axons structures [13]. A significant break-through came in 2015 with the development of a class of convolutional neural networks (CNNs) that use symmetric contractive and expansive passes over augmented data samples. Such an approach allowed for a balance between localization and context, as well as a robustness to deformations and variance within the examples from which the network learned. The first such class of these networks, U-Net, won the ISBI (IEEE International Symposium on Biomedical Imaging) cell tracking challenge in 2015 by a wide margin [14]. This success yielded a plethora of new U-Net architectures and approaches intended to refine and build on this advancement, but also to address some of its limitations. The first limitation is that CNNs require that their hyperparameters be tuned to obtain optimal performance, which can be considerably time consuming. In addition, the problem of having a sufficient number of hand-annotated datasets with which to train the CNN remains an issue.

**Fig 1:**
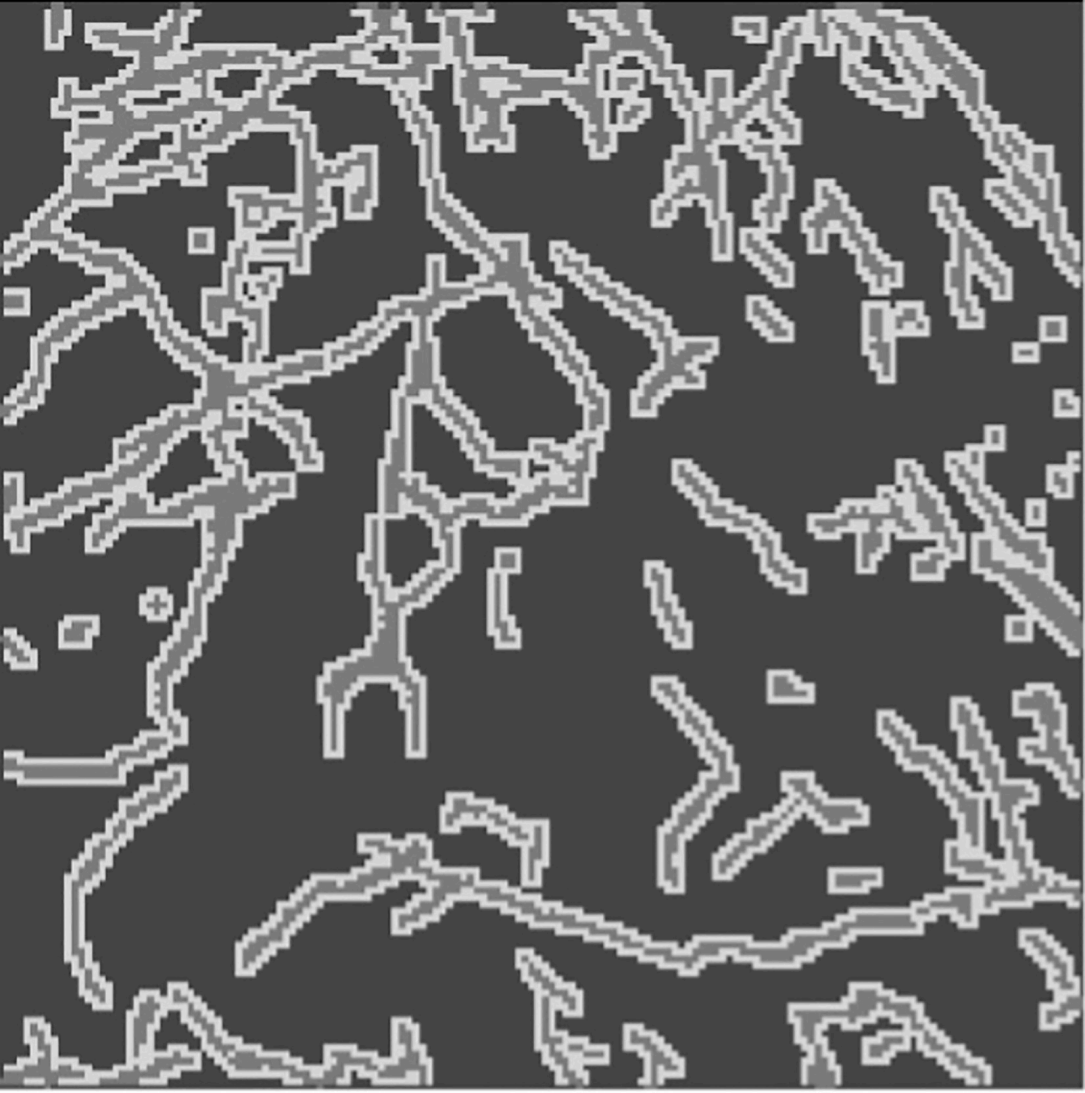
Data label: An expert-labeled slice where darker regions are background, lighter regions are axons, and the lightest regions are edges.

TrailMap (Tissue Registration and Automated Identification of Light-sheet Microscope Acquired Projectomes) is a Python package developed for training a 3D U-net model for axon segmentation by Friedmann et al. [15]. The original TrailMap model was trained with volumes of brain substacks containing labeled serotonergic axons and subsections containing imaging artifacts and background. Each volume contained 3–10 labeled slices (Fig 1 shows an equivalent example from our dataset). The package’s evaluation accounts for these sparsely labeled images by masking unlabeled slices. TrailMap generates a one-pixel boundary representing the edges of axons from the images and devalues their misclassification in calculating loss compared to the misclassification of axons. This ensures that a false positive within one pixel of the axon is not strongly penalized, thereby minimizing the effect of pixel-size differences in expert labeling.

Technologies such as TrailMap contribute to the growing effort to develop templates for integrating data across multiple non-disruptive 3-dimensional image modalities, combining insights from LSFM and MRI into a common coordinate framework [15, 16]. Such co-registration is essential for understanding brain function in both normal and diseased conditions, including delivering brain site-specific drugs and implanting electrodes precisely for recording or stimulation.

Transfer learning can be a useful method in which the features learned from another domain with a richly annotated dataset are used to segment a new smaller dataset outside of the original domain [17]. Fine-tuning the TrailMap model provides an opportunity to exploit the benefits of transfer learning specifically from other images within this domain [15], such as our dataset of labeled brain-wide noradrenergic projections. Indeed, TrailMap includes code and instructions for transfer learning, and the authors already had successfully applied transfer learning to prefrontal cortical projections with 17 training cubes. Our training dataset, however, is much smaller.

We wished to keep the innovative features of TrailMap (e.g., sparse labeling, edges generation, network weights from pretraining), while improving the performance of the fine-tuned model on our dataset. While naturally maintaining the same TrailMap voxel label structure of axons and background, our annotated data involves a different neurotransmitter system (noradrenergic rather than serotonergic), an updated clearing technique, and a different light sheet microscope (LifeCanvas SmartSPIM rather than LaVision Ultramicroscope II light-sheet). Neurons have many diverse dendritic morphologies and axonal projections [18], so one implication of using neurons from a different neurotransmitter system is that the original model could improve its performance within the new neurotransmitter system, which might have different axonal projection types, by being fine-tuned.

In optimizing fine-tuning, we incorporated several principles from nnU-net (no-new-U-Net) as modifications in the TrailMap code [19]. nnU-net is a codebase that decides the optimal training values for some parameters based on the characteristics of a database along with others that remain fixed regardless of the dataset. Many of these fixed parameters used by nnU-net are relevant to fine-tuning models created from other codebases, like TrailMap, including parameters for data augmentation, data foreground sampling, scheduled learning rate, and the inference overlap method. We will refer to modifications inspired by the nnU-net codebase as the “nnU-net modifications” in this paper.

One optimization approach to avoid overfitting is data augmentation. Data augmentation is the process of increasing the amount of training data by generating new instances from existing data; the strategies employed reduce the tendency of a model to overfit. Common techniques include a combination of spatial transformations such as scaling, rotation, reflection, and cropping. TrailMap includes algorithms for rotations and horizontal, vertical, and depth-wise flipping of volumes. We also tested algorithms for elastic deformation as we thought the natural variation in axons might mimic such a transformation. Elastic deformation was not mentioned as an augmentation method in the nnU-net paper, but it is included in the nnU-net codebase [19] and natural variation in axons may also mimic elastic deformations. We did not implement all the augmentation techniques included in nnU-net, including notably, augmentations affecting the intensity of the image by altering the z-score; TrailMap had already determined that z-score normalization, by removing the raw intensity values from the images, reduced the ability of the model to distinguish background from axons.

**Fig 2:**
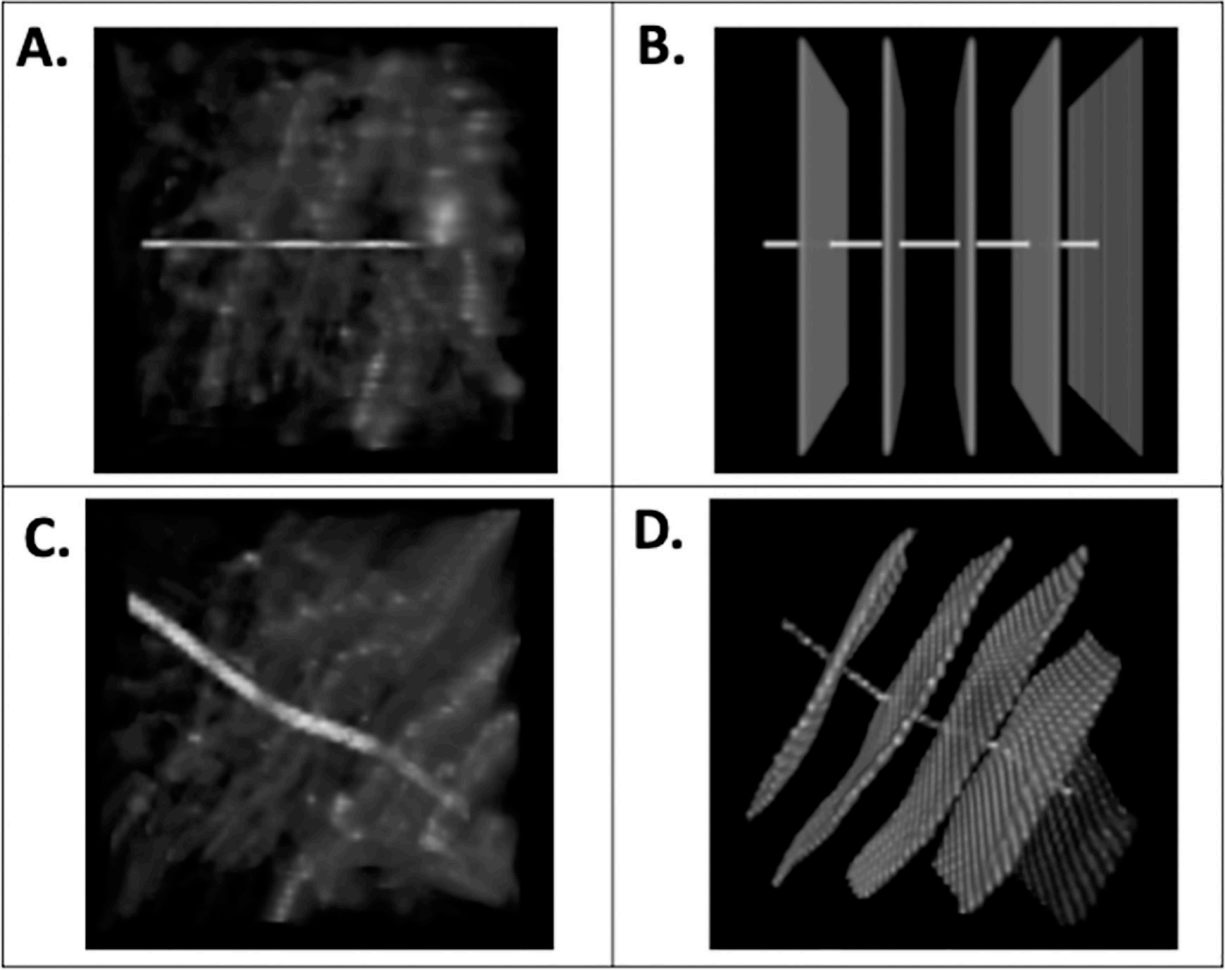
Data Augmentation: A: image inputs (top row) with a horizontal line added for transformation comparison. B: A labeled volume with all labels (axons, edges, and background) in white. C. input transformation D. label transformation. For A and C the intensity is adjusted so 0 is the minimum and 5140 is the maximum (visualized using intensities divided by 65535 and multiplying by 255 within the code, and then adjusted using 0 and 20 as the max and min within Fiji). All images are 186.6 um on the original rotation x and y axis and 200.0 um on the original rotation z axis and displayed with Fiji. Using 100 pixel^3^ cubes for illustration.

An illustration of the spatial transformations on an input volume and labels can be seen in Fig 2. It includes an artificial line to illustrate that the volume inputs and labels remain correctly mapped to each other after the transformation.

An additional nnU-net modification is to over-sample the 3D volumes from areas in the full training image with foreground features [19]. The foreground features for our study are the axons, which are indicated in the labeled data, so we included over-sampling as well, which we labeled as a nnU-net modification.

nnU-net uses a learning rate that decays during training. We added in an adaptive learning rate that decreases when there is a prolonged period of no improvement to the validation loss, which we labeled as a nnU-net modification. The learning rate in a deep learning network controls how quickly the model changes to incorporate the new patterns being presented. A rate that is too low will progress very slowly, however; a rate that is too high can cause unstable behavior. In addition to testing an adaptive learning rate, we also tested using a lower learning rate than was used in the original training.

Finally, nnU-net provides code for utilizing overlapping sliding windows during inference. The overlapped windows predictions are each weighed by a Gaussian matrix the same size as the window. The Gaussian matrix has higher weights at the center of the matrix and lower weights at the edges. The reason that using these overlapping windows should increase the performance is that predictions at the edges of volumes might be less accurate due to edges having less surrounding information then the center of volumes and thus should be weighted less.

An additional parameter we tested was fine-tuning only some layers of the model. When transfer learning with a smaller dataset, it is often best practice to fine-tune only some layers of the existing model to avoid overfitting [20]. Previous studies have investigated which layers of U-net should be trained for optimal performance, and found that training the encoding layers of a U-net model resulted in a higher dice score than training the decoding layers [21]. Since we are using a small training dataset, we tested training only the first, middle, and last two convolutional layers, as well as training all the U-net layers.

Our study endeavored to optimize transfer learning using principles from the nnU-net framework as well as other principles for fine-tuning U-net. We compared the performance in identifying axons in expert-annotated mouse brain slices both before and after fine-tuning our annotated data. We also compared the performance of fine-tuning using the standard settings from the TrailMap package [15] against the TrailMap package with the preprocessing and post-processing modifications suggested by nnU-net [19, 22] and other U-net literature.

## Materials and methods

### Animal experiments

All animal experiments and handling were conducted in accordance with the US National Institutes of Health Guide for the Care and Use of Laboratory Animals and were approved by the Institutional Animal Care and Use Committee (IACUC) of Oregon Health & Science University (#IP00000955). *Dbh*^*Cre*^ mice were generously gifted by Dr. Patricia Jensen at NIHES (also available from The Jackson Laboratory; stock #033951) [23] and maintained by backcrossing with wildtype *C57Bl/6* mice acquired from Charles River, or home-bred within 5 generations of Charles River breeders. Mice 1–3 months old of both sexes with approximately equal proportions were used and were maintained on a 12-hour light-dark cycle, with food and water available ad libitum.

### Stereotaxic viral injections

Injections were performed using our established procedures [24]. Briefly, *Dbh*^*Cre+/-*^ mice at 6 weeks of age were anesthetized with isoflurane (4% induction, 1–2% maintenance) and stabilized in a custom stereotaxic apparatus with body temperature being maintained by a heating pad. A small craniotomy was made over each target of interest and a pulled glass micropipette (Drummond, tip diameter: 15–20 μm), beveled sharp and loaded with the injectant, was lowered into the brain. Pipettes were front-filled with AAV (serotype 2/1) expressing either CAG-FLEX-GFP (UPenn, lot# V0827) or pCAG-FLEX-tdTomato-WPRE (Addgene cat# 51503). 3 min after reaching the target, 30–50 nL of virus was dispensed using a hydraulic injector (Narishige) followed by a 5-min waiting period. The pipette was retracted 0.3 mm, paused for 10 min, and then fully retracted. Up to two injections were made per mouse targeting the locus coeruleus bilaterally (in mm relative to Bregma; 4.80 posterior, +/- 0.75 lateral, 4.00 ventral).

### Tissue collection

Three (3) weeks after viral infection, mice were perfused transcardially with 50 mL room-temperature (RT) phosphate-buffered saline (PBS) followed by 50 mL of ice-cold 4% paraformaldehyde (PFA, w/v) and an additional 30 mL of RT PBS. The extracted brain was post-fixed in 4% PFA at 4°C overnight under gentle agitation, rinsed 3x in RT PBS for 1 h under agitation, and then finally stored in PBS with 0.02% NaN_3_ at 4°C.

### Tissue dilapidation, labeling, and clearing

Fixed brains were processed using a recent optimization of the AdipoClear framework (protocol v1.0 from https://mab3d-atlas.com) [25, 26]. All steps used gentle agitation at RT unless stated otherwise. Samples are first washed 3x (2 h, 4 h, overnight) in B1n buffer (in H_2_O: 0.1% Triton X-100, 2% glycine, 0.01% 10N NaOH, 20% NaN_3_) to block excess PFA and preserve antigens. Next, samples were delipidated with SBiP buffer (200μM Na_2_HPO_4_, 0.08% sodium dodecyl sulfate, 16% 2-methyl-2-butanol, 8% 2-propanol in H_2_O (pH 7.4)) for 1 h, 2 h, 4 h, overnight, and 3x 1 d. Samples were then rehydrated overnight in B1n buffer, then rinsed 4x (1 h, 2 h, 4 h, overnight) in PBS w/ NaN_3_. To begin immunolabeling, brains were first blocked with PTxwH buffer (in PBS: 0.1% Triton X-100, 0.05% Tween-20, 0.002% w/v heparin, 20% NaN_3_) for 4x (1 h, 2 h, 4 h, overnight). Samples were then incubated with primary antibodies (rabbit polyclonal anti-RFP, Rockland cat# 601-401-379, lot# 42872, 1:500; goat polyclonal anti-GFP, Rockland cat# 600-101-215, lot# 35577, 1:500) diluted in PTxwH for 20 d at 37°C, then rinsed 5x (1 h, 2 h, 4 h, 12 h, 24 h) in PTxwH at RT. Next, samples were incubated with secondary antibodies (donkey polyclonal anti-rabbit Alexa 594, Jackson Immunoresearch cat# 711-587-003, lot# 148972, 1:150; donkey polyclonal anti-goat Alexa 647, Jackson Immunoresearch cat# 705-607-003, lot# 150295, 1:150) diluted in PTxwH for 18 d, and rinsed 5x (1 h, 2 h, 4 h, 12 h, 24 h) in PTxwH. Samples were then bleached in 0.3% H_2_O_2_ at 4°C overnight, and washed 3x in 20 mM PB (16 mM Na_2_HPO_4,_ 4 mM NaH_2_PO_4_ in H_2_O) at RT for 2 h. For further delipidation, samples were dehydrated in an ascending gradient (20%, 40%, 60%, 80%) of MeOH in H_2_O for 1 h each, 3x (1 hours, 1.5 hours, 2 hours) 100% MeOH, then a descending gradient (80%, 60%, 40%, 20%) of MeOH in H_2_O for 1 h each. Next, brains were washed twice (2 h, 4 h) in 20 mM PB and finally twice in PTS solution (25% 2,2’-thiodiethanol/10 mM PB) (2 h, overnight), then equilibrated with 75% histodenz buffer (Cosmo Bio USA AXS-1002424) with refractive index adjusted to 1.53 using 2,2’-thiodiethanol. Samples were stored at ~20°C until acquisition.

### Light-sheet microscopy

The cleared brain samples were imaged horizontally with tiling using the LifeCanvas SmartSPIM light-sheet microscope. 561/647 nm lasers were used for Alexa 594/647 imaging with the 3.6×/0.2 NA detection lens. Light-sheet illumination was focused with NA 0.2 lenses from each side, and axially scanned with an electrically tunable lens coupled to the sCMOS camera (Hamamatsu Orca Back-Thin Fusion) in slit mode. The camera exposure was set at fast mode (2 ms) with 16-bit imaging. Image stacks were acquired along the dorsoventral axis following a 4 x 6 grid configuration. The x/y sampling rate was 1.866 μm and z step at 2 μm. Individual images from each stack originating from the same z-plane were stitched together using the TeraStitcher module (https://abria.github.io/TeraStitcher/) [27] resulting in ~3600 horizontal image planes spanning the whole brain.

### TrailMap imaging

**Fig 3:**
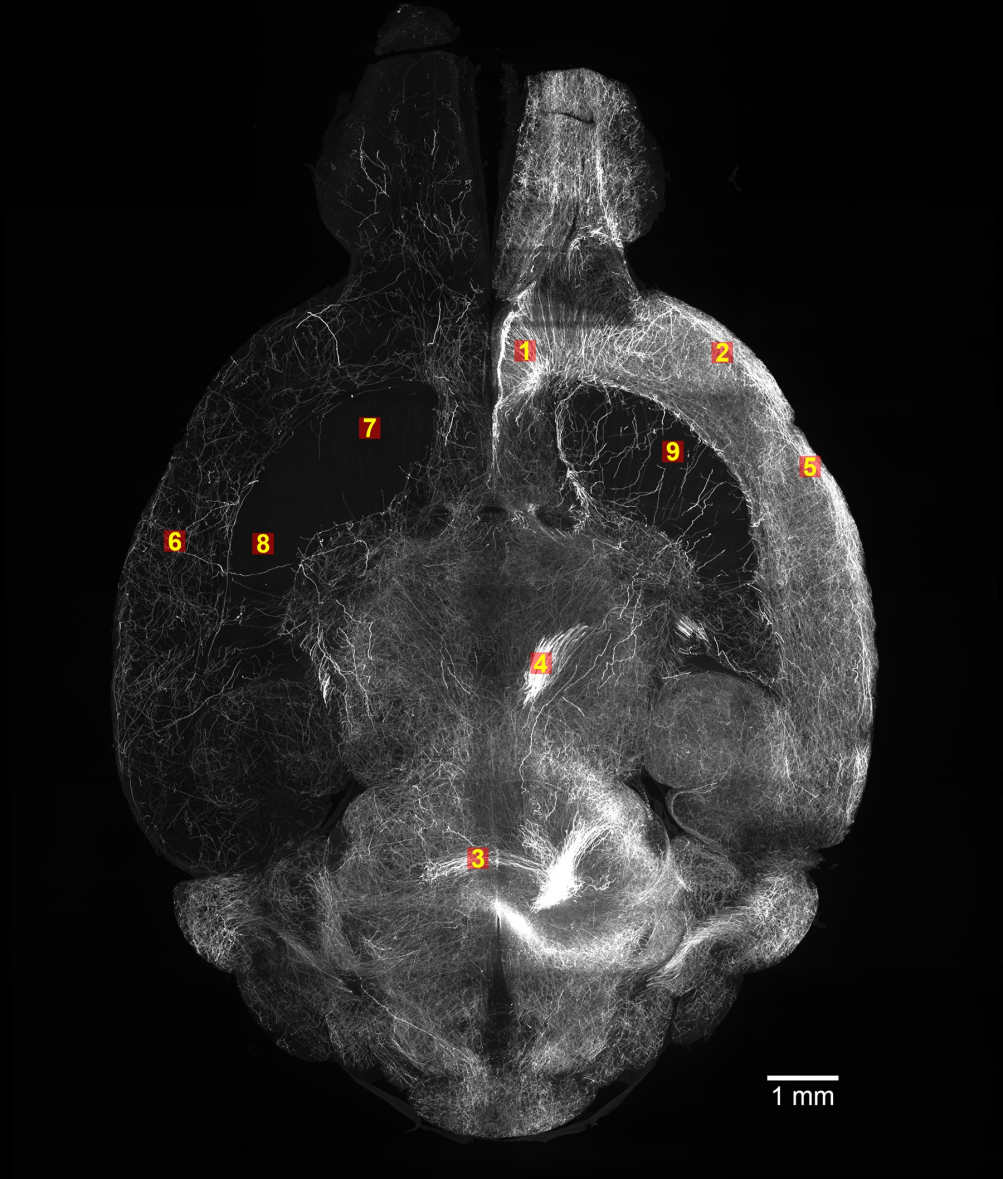
Location of labeled data. Nine 160 pixel^3^ labeled cubes were available in total.

The labeled image cube subsets used in this study are 160 pixels in the x, y, and z direction (~ 320 x 320 x 300 μm^2^), with corresponding 188 pixel^3^ padded source image input cubes. All cubes originated from the same set of horizontal sections (~4.5 mm below dorsal origin) and were selected at x/y positions that captured differing densities or “textures” of axonal labeling for training (Fig 3). For each cube, every 20th section is annotated manually, starting at the 15th slice.

For expert annotation, in Fiji, an image processing package, we enhanced the contrast with these settings: saturated: 0.35%, normalized: true, equalize: false, process all slices: true, use stack histogram: true, and exported the stack for loading in TrakEM2 [28]. In TrakEM2, a modeling program, we applied the CLAHE filter with settings: blockRadius: 63, bins: 255, slope: 3.0, and fast: true [29]. Sections were annotated using the AreaList interface and exported as binary label images.

After the expert annotation, the label images were processed with TrailMap’s axon edge annotator script to provide the edge labels. These images were subsequently used for training, validation, and testing of the modified TrailMap model.

### TrailMap fine-tuning

We used the original TrailMap codebase from GitHub (https://github.com/AlbertPun/TRAILMAP) with our modifications [15]. We loaded the original pretrained model from TrailMap and fine-tuned it with our dataset using 50 epochs with batch-size of 6. Nine 160 pixel^3^ labeled cubes were available in total, with corresponding 188 pixel^3^ input cubes (Fig 3). The data included three densely populated cubes (1, 2, and 5), two moderately populated cubes (3 and 4), and one sparsely populated cube (6). We trained each model with eight cubes six times for each different parameter configuration. For each of the six trainings, we left out a different cube with labeled axons for testing. The cubes were divided such that training and validation sample volumes were created from separate areas of the cube–the validation data was created from the last 64 pixels along the x axis, while the training data was created from the remaining cube section. We used 75 3D volume samples from each cube for training and 25 samples for validation, each with a 64-pixel^3^ input dimension and 32-pixel^3^ output dimension. The smaller size of the output is a feature of the TrailMap code–the model predicts axons only for the centers of the input volumes to make use of pixels around of the predicted area. The volumes are generated by a TensorFlow generator during the training.

For each epoch, the number of validation steps was the number of volumes (600 for training, 200 for validation) divided by the step size (6). We used an Adam optimizer and cross-entropy as the loss function, as used in the original TrailMap training scheme. The loss function weighted the classes for the axons, backgrounds, artifacts, and edges as 1.5, 0.2, 0.8, and 0.05, respectively. We tested using 0.5 and 1.0 weights for the background class as well as equal weights for all classes. The model was saved during training at the validation loss minimum.

### Data augmentation

The nnU-Net augmentations were implemented by incorporating the “augment_spatial” function from Batchgenerators [30]. TrailMap includes code for rotations and horizontal, vertical, and depth-wise flipping of volumes and rotations. Due to ease of use with elastic deformation, we used the Batchgenerators code for both rotations and elastic deformation but used the TrailMap code for flipping.

The intensity was divided by a maximum intensity value for training and validation data. We continued using the TrailMap method of intensity augmentation in training data, where the data was randomly scaled and summated with a random constant. Unlike in TrailMap, we did not augment the validation data to align with the common practice of only augmenting training data.

### Foreground over-sampling

The nnU-net code for oversampling randomly over-samples from the “foreground” data instead of all the data [19]. The foreground data in TrailMap are labeled as axons. Within nnU-net, if a randomly selected volume extends past the limits of the cube, the volume is padded. With our input cubes sized at 188-pixel^3^ and volumes sized at 64-pixel^3^, this method would result in a high percentage of padded data. Instead, we continued to use the TrailMap sampling method, where the top, left, back corner of the training volume is selected randomly from the full image to create the training volumes. The top-left-back corner is selected from an area with at least one training-volume dimension (64 pixels) from the opposite edges to ensure the cropping will produce a 64-pixel^3^ training volume. When oversampling axons, we selected the top, left, back corner pixels from a list of axon-labeled pixels 30% of the time.

### Learning rate

nnU-net uses a learning rate with a polynomial decay with 1000 epochs and a starting learning rate of 0.01. Since we are fine-tuning rather than training, and only using 50 epochs and a lower starting learning rate, we tested reducing the learning rate by a factor of 0.1 only after a metric has stopped improving with a built-in TensorFlow learning-rate scheduler, ReduceLROnPlateau [31], as a nnU-net modification. TrailMap had a learning rate of 1e-3, but we also tested lower learning rates of 1e-4 and 5e-4.

### Trainable layers

We tested fine-tuning only the first, middle and last two 3D convolutional layers, as well as training with all the layers. When fine-tuning two layers, we did not freeze the batch-normalization layer.

### Gaussian weighted overlapping windows

Our modified TrailMap code implemented the same logic as nnU-net and incorporated some of nnU-net’s Gaussian-related functions to calculate the value of overlapping windows. However, we did not extend the Gaussian matrix function with a Gaussian prediction for mirrored images as implemented in nnU-net [19]. In the Gaussian matrix function, each overlapping volume is multiplied by a Gaussian matrix before being added to the predicted image. Each resulting predicted value is then divided by the sum of the Gaussian multipliers used at that location in the image. The final value at each voxel is a weighted sum, with the weights representing the centrality of the voxel of each volume.

### Performance metrics

For the performance metrics in this paper, the TrailMap inference function was used to predict axons for the validation and test data cubes. The TrailMap inference function segments the image in a sliding window. The input image used by TrailMap is larger than the segmented output (64 pixel^3^ input vs. 36 pixel^3^ output), so the segmented image is smaller than the input image by an offset at the edges (64 pixel^3^ input– 36 pixel^3^ output/2 = 14-pixel offset). Therefore, we used a 188 pixel^3^ input to predict a 160 pixel^3^ output. The performance metrics were obtained from the labeled input and the segmented output for both the validation and test datasets.

The metrics used were:

$$Accuracy=\frac{TP+TN}{TP+TN+FP+FN}$$

$$Precision=\frac{TP}{TP+FP}$$

$$Edge\;Precision=\frac{TP}{TP+FP-EA}$$

$$Recall=\frac{TP}{TP+FN}$$

$$F1\;Value=\frac{2*Recall*Precision}{Recall+Precision}$$

$$Edge\;F1\;Value=\frac{2*Recall*Edge\;Precision}{Recall+Edge\;Precision}$$

with Edges identified as Axons (EA), True Positive (TP), True Negative (TN), False Positive (FP), and False Negative (FN).

The authors of TrailMap noted that they did not include edges when calculating performance metrics [15]. To remain consistent with the original code, we also calculated “Edge Precision” and “Edge F1-score”, where the edges classified as axons were not included in the false positives. We used Edge F1-score as our primary performance evaluator so both recall and precision would be considered. Using just the recall would weigh the False Negatives while disregarding any False Positives, and using only precision would weigh the False Positives while disregarding any False Negatives.

Each modification combination has three calculations–the overall average metrics and the overall average and standard deviation of the difference between the metrics of the models trained with modifications vs. the models trained with a default modification. The first, the overall average, is calculated per unique modification combination by taking the overall average of the average metrics per validation section, with each validation section inferred by the five models trained using that validation section during validation. We also calculated the difference between the average metrics resulting from using models trained with a default modification and the average metrics with the new modifications for each validation section. We calculated the average and standard deviation of the differences from the default models for each unique modification combination across all validation sections. We only calculated the metrics for cubes that included axons (cubes 1–6). The overall average for the testing dataset was calculated by averaging the metrics for each test cube, inferred with the model which did not use that cube for training or validation. We also calculated the average and standard deviation of the differences between the metrics resulting from using the fine-trained model vs. the original model over all test cubes.

## Results

After training, we segmented the validation datasets to determine the optimal modifications to the TrailMap code[15]. Since the default settings were selected with a much larger training set while training the original TrailMap, we did not want to change any defaults unless the change resulted in a general Edge F1 score improvement across validation sections. Therefore, for each modification type (class weights, starting learning rate, trainable layers, nnU-net modifications), we only continued to use modifications that increased the difference of the edge F1 score from the default modification by at least half of the standard deviation. After selecting the best modifications, we compared the performance of both the best fine-tuned TrailMap model and the original model with the test datasets.

### Weighing loss function

The performance metrics of the model after fine-tuning with a background weight of 0.2, 0.5, 1.0, and 1.5 for loss are shown in Table 1. With the background weight of 1.5, all the classes have an equal weight. The background weight of 1.0 improves the Edge F1 score by more than half of the standard deviation, so we will use that background weight moving forward.

**Table 1:** Performance metrics with a background class weight of 0.2, 0.5, 1.0, and 1.5 for loss. With the background weight of 1.5, all the classes have an equal weight. The Std Dev Diff represents the standard deviation over validation sections of the differences of the metrics to the default background weight of 0.2 and the Avg Diff is the overall average differences compared to the default model.

Background weight	Adjusted Accuracy	Axon Precision	Edge Axon Precision	Axon Recall	F1 Score	Edge F1 Score
**0.2 Default**	**0.898**	**0.516**	**0.794**	**0.941**	**0.657**	**0.855**
**0.5**	**0.907**	**0.544**	**0.830**	**0.920**	**0.675**	**0.867**
Avg Diff	0.010	0.028	0.036	-0.020	0.018	0.012
Std Dev Diff	0.006	0.006	0.012	0.011	0.005	0.010
**1.0**	**0.918**	**0.582**	**0.868**	**0.894**	**0.695**	**0.875**
Avg Diff	0.020	0.066	0.074	-0.046	0.037	0.020
Std Dev Diff	0.013	0.019	0.024	0.027	0.010	0.027
**Equal**	**0.952**	**0.806**	**0.954**	**0.685**	**0.730**	**0.790**
Avg Diff	0.054	0.291	0.160	-0.256	0.072	-0.065
Std Dev Diff	0.040	0.040	0.070	0.072	0.082	0.088

### Freezing layers

The performance metrics of the model after fine-tuning all the layers or the first, middle, or last two convolutional layers of the neural network on the validation dataset are shown in Table 2. None of the new frozen layers led to an improvement of over half of the standard deviation so we continued to use the default of full layer training.

**Table 2:** Performance metrics with 1) all U-net layers trainable (Full), or 2) the first two layers (First), 3) the middle two layers (Middle), or 4) the last two layers (Last) (decoder) trainable, with the validation dataset. The Std Dev Diff represents the standard deviation over validation sections of the differences of the metrics to the default Full Layer Trainable and the Avg Diff is the overall average differences compared to the default model. All models had a background weight of 1.0.

Trainable Layers	Adjusted Accuracy	Axon Precision	Edge Axon Precision	Axon Recall	F1 Score	Edge F1 Score
**Full Layer (Default)**	**0.918**	**0.582**	**0.868**	**0.894**	**0.695**	**0.875**
**First Layer**	**0.907**	**0.542**	**0.841**	**0.927**	**0.678**	**0.878**
Avg Diff	-0.011	-0.040	-0.027	0.033	-0.017	0.003
Std Dev Diff	0.009	0.022	0.015	0.026	0.012	0.021
**Middle Layer**	**0.908**	**0.548**	**0.799**	**0.862**	**0.659**	**0.820**
Avg Diff	-0.010	-0.034	-0.069	-0.032	-0.036	-0.055
Std Dev Diff	0.006	0.025	0.052	0.051	0.020	0.037
**Last Layer**	**0.915**	**0.582**	**0.875**	**0.894**	**0.696**	**0.879**
Avg Diff	-0.003	0.000	0.007	-0.001	0.001	0.004
Std Dev Diff	0.013	0.040	0.046	0.021	0.027	0.022

### Modifying learning rate

The performance metrics of the model with a learning rate of 1e-3 (the default), 5e-4, or 1e-4 and the validation dataset are shown in Table 3. None of the new learning rates lead to an improvement of over half of the standard deviation so we continued to use the default of 1e-3.

**Table 3:** Performance metrics with the 1e-3 (Default), 5e-4, and 1e-4 learning rate. The Std Dev Diff represents the standard deviation over validation sections of the differences of the metrics to the default learning rate of 0.001 and the Avg Diff is the overall average differences compared to the default model. All models had a background weight of 1.0 and were trained with the full layer.

Learning Rate	Adjusted Accuracy	Axon Precision	Edge Axon Precision	Axon Recall	F1 Score	Edge F1 Score
**0.001 (Default)**	**0.918**	**0.582**	**0.868**	**0.894**	**0.695**	**0.875**
**0.0001**	**0.915**	**0.565**	**0.861**	**0.903**	**0.689**	**0.878**
Avg Diff	-0.003	-0.017	-0.007	0.009	-0.006	0.003
Std Dev Diff	0.005	0.027	0.011	0.027	0.008	0.016
**0.0005**	**0.920**	**0.594**	**0.882**	**0.880**	**0.698**	**0.875**
Avg Diff	0.002	0.012	0.014	-0.014	0.003	0.000
Std Dev Diff	0.004	0.009	0.013	0.015	0.010	0.013

### nnU-net modifications

The results for single modifications are shown in Table 4. The nnU-net modifications used were: over-sampling axons, using an adaptive learning rate, using elastic deformation, and using rotations, in addition to the built-in TrailMap augmentation of flipping the volumes. The rotation augmentation most improved the average Edge F1.

**Table 4:** Performance metrics for models with elastic deformation (Elastic), rotation (Rotate), flipping (Flip), learning rate scheduler (Learning rate), and over-sampling for the validation dataset. The Std Dev Diff represents the standard deviation over validation sections of the differences of the metrics to the default of No Augmentation and the Avg Diff is the overall average differences compared to the default model. All models had a background weight of 1.0, all layers fully trainable, and a 0.001 learning rate.

	Adjusted Accuracy	Axon Precision	Edge Axon Precision	Axon Recall	F1 Score	Edge F1 Score
**No Augmentation (Default)**	**0.918**	**0.582**	**0.868**	**0.894**	**0.695**	**0.875**
**Elastic**	**0.917**	**0.574**	**0.876**	**0.918**	**0.701**	**0.893**
Avg Diff	-0.001	-0.008	0.008	0.023	0.006	0.018
Std Dev Diff	0.007	0.037	0.024	0.041	0.016	0.019
**Rotate**	**0.923**	**0.602**	**0.905**	**0.904**	**0.715**	**0.900**
Avg Diff	0.005	0.020	0.038	0.010	0.020	0.025
Std Dev Diff	0.007	0.053	0.043	0.061	0.019	0.015
**Flip**	**0.919**	**0.595**	**0.901**	**0.891**	**0.703**	**0.889**
Avg Diff	0.001	0.013	0.034	-0.003	0.008	0.014
Std Dev Diff	0.010	0.067	0.060	0.079	0.023	0.019
**Learning Rate**	**0.919**	**0.581**	**0.875**	**0.889**	**0.695**	**0.877**
Avg Diff	0.001	-0.001	0.007	-0.005	0.000	0.002
Std Dev Diff	0.002	0.012	0.006	0.006	0.007	0.005
**Oversample**	**0.916**	**0.576**	**0.864**	**0.895**	**0.692**	**0.873**
Avg Diff	-0.002	-0.006	-0.003	0.000	-0.003	-0.001
Std Dev Diff	0.006	0.018	0.020	0.010	0.012	0.008

We also tested using all the modifications that improved the Edge F1 Score by more than half the standard deviation, but using all the modifications did not improve the model compared to using just the rotation augmentation as shown in Table 5.

**Table 5:** Performance metrics for the validation dataset for models trained with all modifications except for over-sampling (Combined changes) and with just the rotation modification (Rotate). The Std Dev Diff represents the standard deviation over validation sections of the differences of the metrics to the default rotate augmentation and the Avg Diff is the overall average differences compared to the default model. All models had a background weight of 1.0, all layers fully trainable, and a 0.001 learning rate.

	Adjusted Accuracy	Axon Precision	Edge Axon Precision	Axon Recall	F1 Score	Edge F1 Score
**Rotate**	**0.923**	**0.602**	**0.905**	**0.904**	**0.715**	**0.900**
**Combined Changes**	**0.909**	**0.558**	**0.874**	**0.932**	**0.690**	**0.897**
Avg Diff	-0.014	-0.044	-0.031	0.028	-0.025	-0.003
Std Dev Diff	0.011	0.026	0.021	0.011	0.022	0.019

### Best overall model

We compared the performance of the best fine-tuned TrailMap model with the original model on the held-out test set. The best model was trained with a 1.0 background weight and rotation augmentation. Table 6 shows that the best fine-tuned TrailMap model had a higher Edge F1-score than the original model (0.8153 vs. 0.7807). S1 Table shows the metrics for each test cube.

**Table 6:** Performance metrics on the test datasets for the best model, which is trained with 1.0 background weights and rotation augmentations, compared with the original model without fine-tuning. The Std Dev Diff represents the standard deviation of the differences of the metrics with the original model and the Avg Diff is the average differences compared to the original model. The trained model had a 1.0 background weight, all the layers trainable, 0.001 learning rate, and rotation augmentation (Rotate).

	Adjusted Accuracy	Axon Precision	Edge Axon Precision	Axon Recall	F1 Score	Edge F1 Score
**Original**	**0.9055**	**0.5732**	**0.7942**	**0.7886**	**0.6516**	**0.7807**
**Rotate**	**0.9111**	**0.6231**	**0.8509**	**0.8304**	**0.6772**	**0.8153**
**Avg Diff**	0.0056	0.0499	0.0567	0.0419	0.0256	0.0346
Std Dev Diff	0.0289	0.0843	0.082	0.1352	0.0572	0.0806

**Fig 4:**
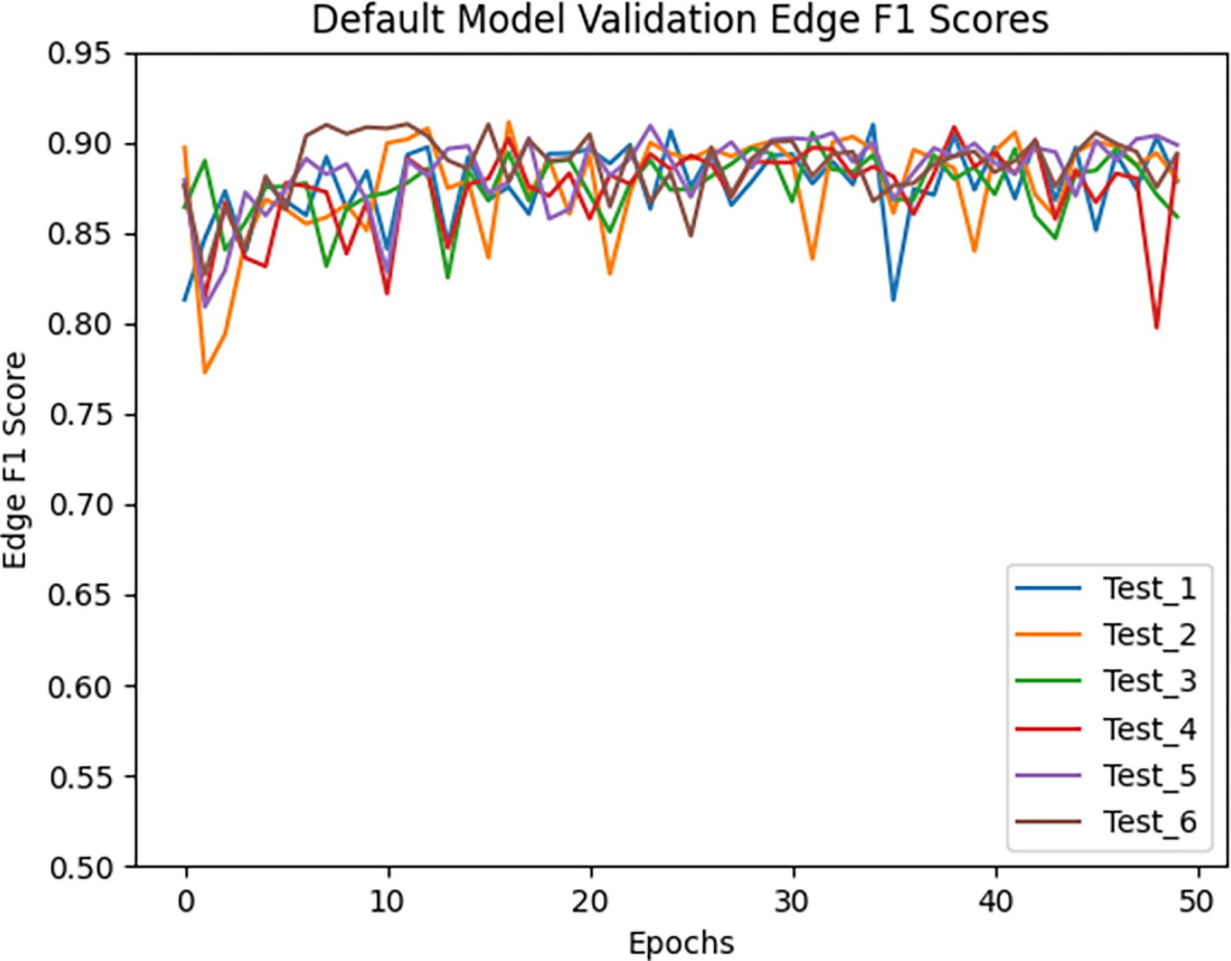
Validation Edge F1 score for the models trained with the default parameters.

**Fig 5:**
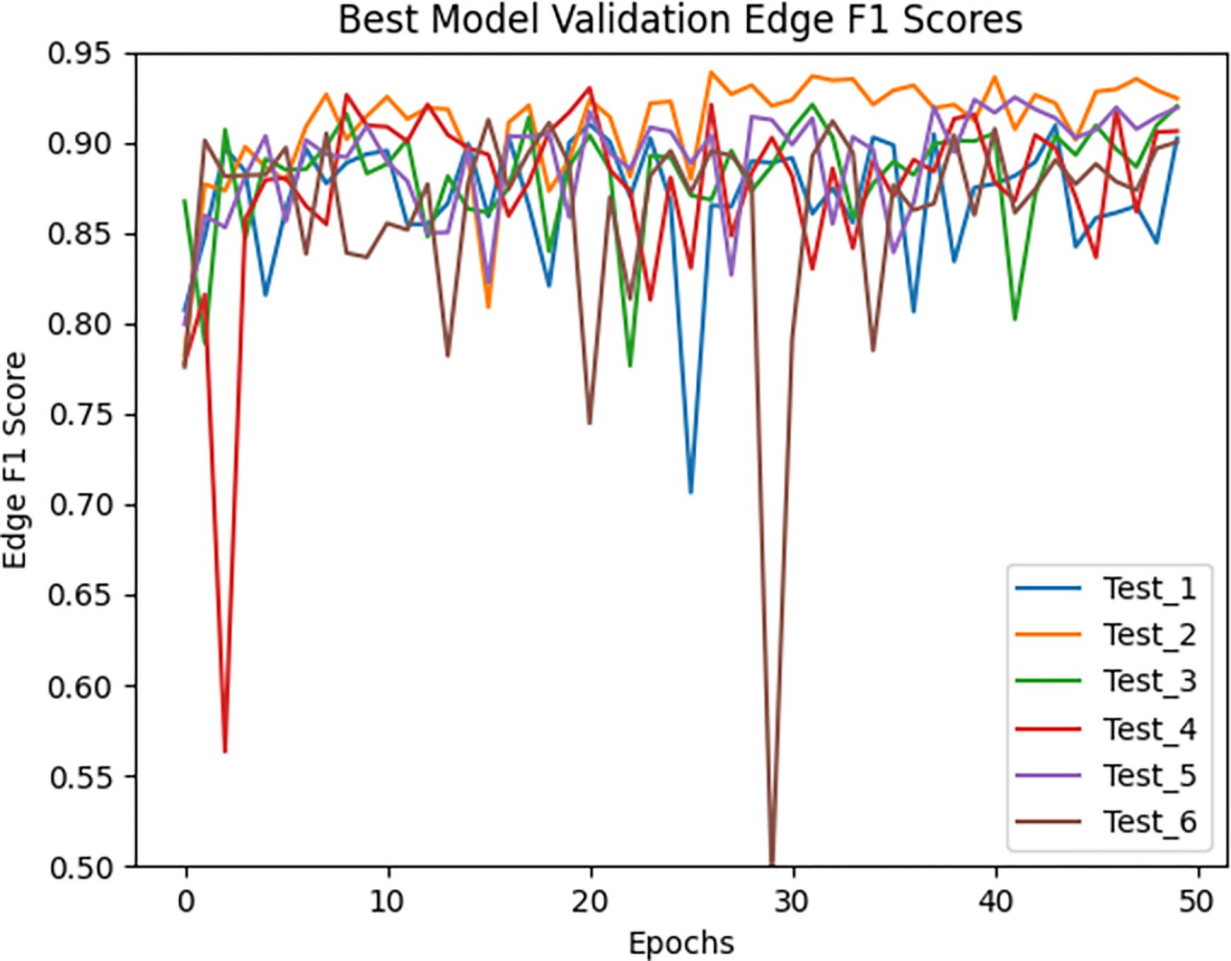
Validation Edge F1 score for the models trained with 1.0 background weight, all the layers trainable, 0.001 learning rate, and rotation augmentation (Rotate).

**Fig 6:**
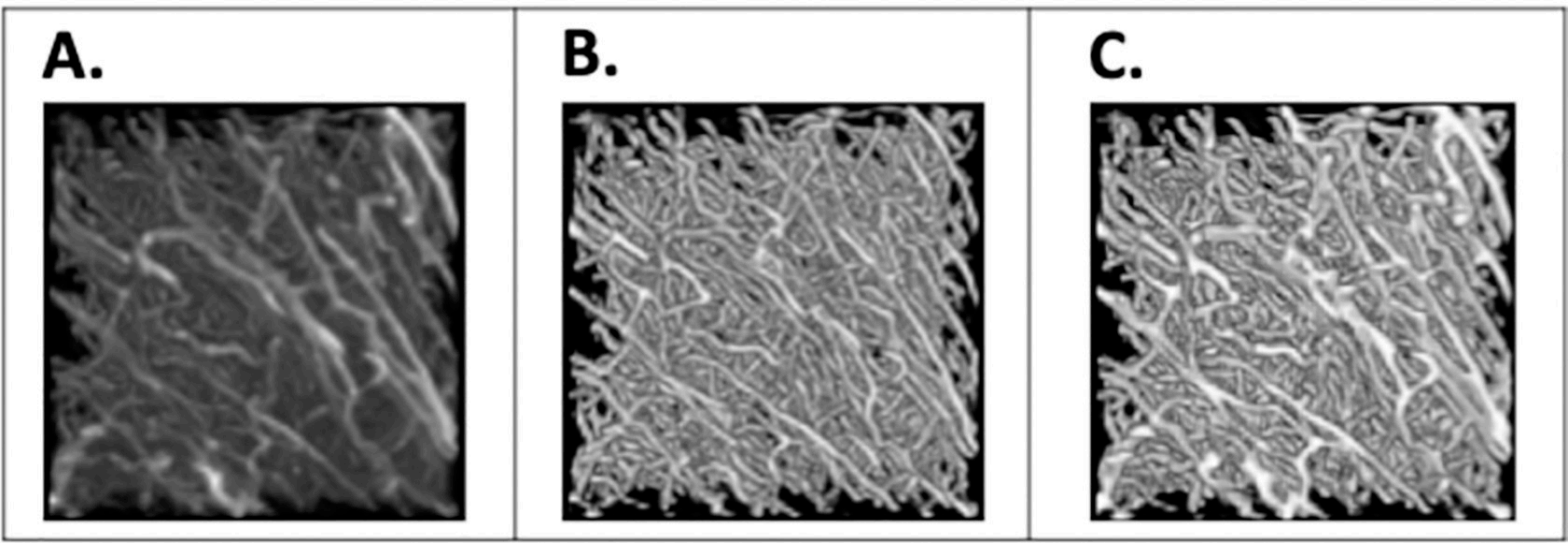
Data Inference results where A) is the input image, B) is the segmentation image result produced using the original model, and C) is the segmentation image result produced using the best fine-tuned model for the Cube 1 test dataset. The input image was processed by the CLAHE filter as described in the methods from Cube 1. Only segmentation values above 0.5 are shown. Displayed with Fiji. The dimensions are 298.56 um on x and y axis and 320 um on the z axis.

**Fig 7:**
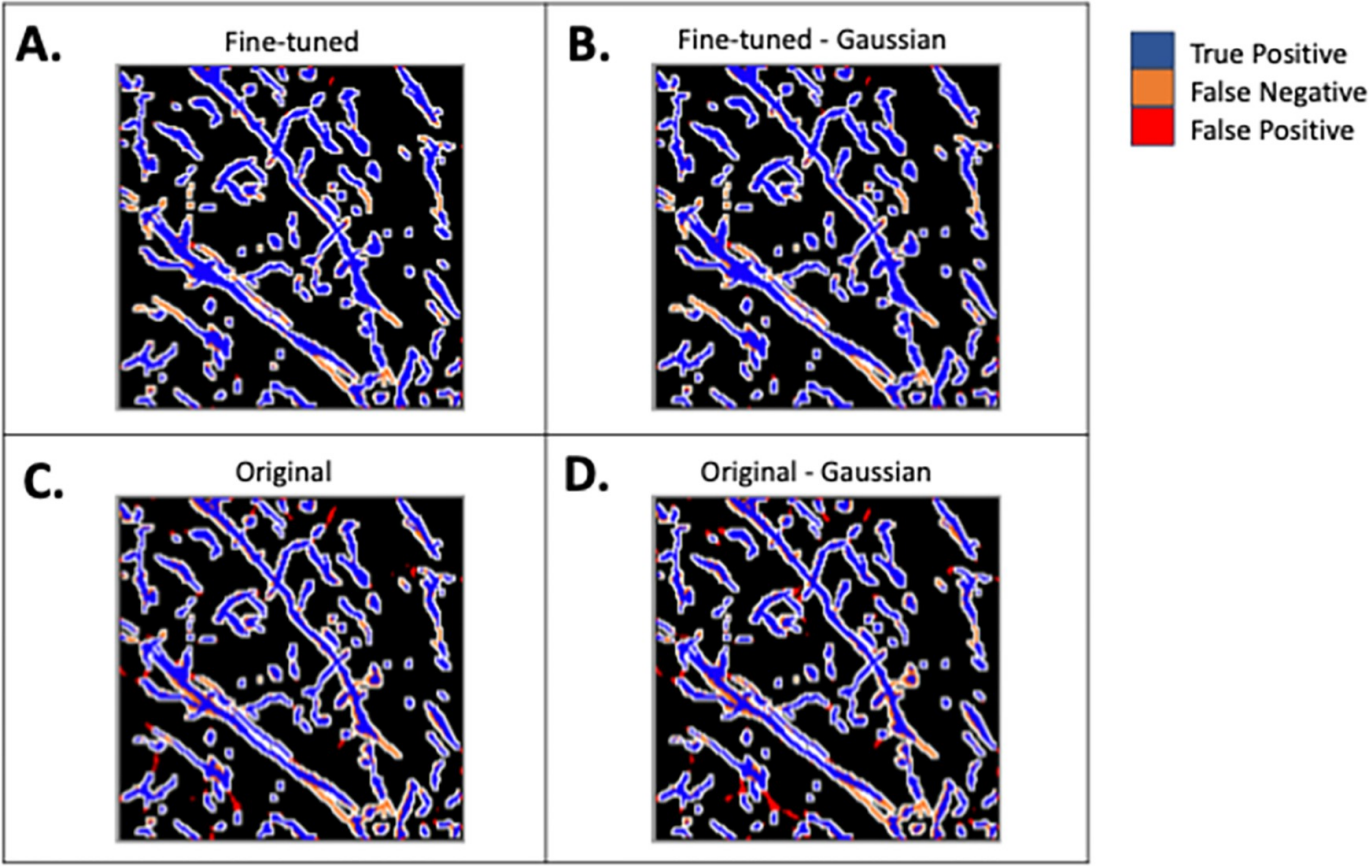
Cube 1 test dataset slice predictions created with A) best fine-tuned model, B) best fine-tuned model with Gaussian overlap inference, C) best original model, and D) best original model with Gaussian overlap inference. The dimensions are 298.56 um on x and y axis.

Figs 4 and 5 show the validation loss for the models trained with the default parameters and the best modifications, respectively. A visualization of the predictions of the original and best TrailMap model (Fig 6B and 6C) is qualitatively like the input image (Fig 6A). Fig 7 shows the color-coded true positives, false positive, and false negatives of the predictions of each model, with and without Gaussian overlap for inference for one labeled slice from Cube 1. The ground truth is represented by the true positives and false negatives.

### Gaussian-weighed overlap

Table 7 indicates if using Gaussian-weighed overlapping windows increases or decreases the metrics. Using a Gaussian-weighed overlap for inference, where the Gaussian matrix weights each voxel by the centrality of the voxel within the current window, improves the F1 Edge score in 9 out of 12 models. Fig 7 shows inference created with Gaussian-weighted overlapping windows in panels C and D. In S2 Table, we showed the differences per test cube between the model inference with and without Gaussian.

**Table 7:** Effect of using Gaussian (G) on all results The Std Dev Diff represents the standard deviation of the differences of the metrics with Gaussian overlap and without the Gaussian overlap and the Avg Diff is the average differences with Gaussian metrics (with a positive value indicating an average improvement with Gaussian overlap). The Original and Rotate columns show the metrics for the original and fine-tuned model without the Gaussian overlap.

	Adjusted Accuracy	Axon Precision	Edge Axon Precision	Axon Recall	F1 Score	Edge F1 Score
**Original**	**0.9055**	**0.5732**	**0.7942**	**0.7886**	**0.6516**	**0.7807**
**Avg Diff**	0.0009	0.0049	0.0026	0.0037	0.0045	0.0038
**Std Dev Diff**	0.0009	0.0083	0.0026	0.0026	0.0031	0.0016
Rotate	**0.9111**	**0.6231**	**0.8509**	**0.8304**	**0.6772**	**0.8153**
**Avg Diff**	0.0000	0.0015	-0.0002	0.0016	0.0009	0.0011
**Std Dev Diff**	0.0009	0.0083	0.0026	0.0026	0.0031	0.0016

## Discussion

Often, the most challenging aspect of fine-tuning an LSFM segmentation model is creating the training data. The labeling process is arduous and requires expert skill to identify the axons. The methods explored here provide a broad roadmap for users to fully utilize and improve upon existing trained models, reducing the burden of manual data annotations. In addition to the modifications to the TrailMap code, we have provided a new dataset of expert-annotated noradrenergic axons. The utility of TrailMap in axon segmentation has generalized efficiently to our dataset both with the original TrailMap model and with the use of fine-tuning and additional modifications. Recently, others have shown success in training a model with nnU-net to segment axons without manual annotation showing state-of-the-art performance [32]. While this new, automated strategy significantly reduces manual input in axon segmentation, the model is still reliant on extensive training data or transfer learning techniques, such as those described in this paper, to develop new training modules for specific axon types or sample batches.

Friedmann et al. had previously successfully fine-tuned the model with 17 training cubes, rather than the 8 that we used [15]. We ultimately show that it is possible to have a positive effect on the model performance even with a smaller training dataset. The average Edge F1 score increased by 0.0346 with a standard deviation of 0.0806.

However, this increase is less than half of the standard deviation due to Cube 6, the only cube with a lower Edge F1 score after fine-tuning (S1 Table). Cube 6 is the only sparsely populated cube, so this shows that the training only improves the model when the test data has similar axon density as the training data. In general, the fine-tuned models themselves might not be generalizable beyond the brain region and imaging processing used during training. The value in this paper is both as a guide for other users wishing to fine-tune the TrailMap model and as a guide for fine-tuning any existing model with a very small amount of data.

Changing the background weight to 1.0 increased the Edge F1 scores, but lowering the learning rate and the trainable layers only resulted in a small increase that was less than half of the standard deviation. Friedmann et al. mention that they chose the initial weights to balance the false positives and negatives and deal with class imbalance. Since our dataset likely has a different background to axons ratio than the original TrailMap training data, it makes sense that we would need to experiment with different weights to optimize the Edge F1 score. As recommended in TrailMap’s extended ReadMe, we did experiment with different augmentations before changing the cross-entropy weights, but the validation Edge F1 score remained as instable as when the model was trained with the default background weight and default augmentations. Previous studies have found that training the encoding layers (the first layers) of the model had the most positive effect, so our results are inconsistent with the results in Amiri et al. (2019), which may be due to the different image types and dataset size used in their fine-tuning training set compared to ours. Also, both lowering the learning rates and freezing the trainable layers did sometimes lead to an improvement, albeit with a much larger standard deviation, so perhaps with a less variable dataset we would have found that these modifications resulted in an average difference in the Edge F1 score greater than the standard deviation of the difference.

The nnU-net modifications all increased the Edge F1 score with the exception of over-sampling. Two possible reasons are that our oversampling method restricts the possible locations of the corners of the volumes to one of labeled slices rather than all slices of data. Another is that in our dataset, we already have a high percentage of axons, so over-sampling might be unnecessary. However, when we tested combining all the nnU-net modifications together except for oversampling, we found that rotation augmentation performed better. Generally, the augmentation of the dataset should mimic the variability of the test dataset. It is possible that the small size of our testing dataset (one cube) does not contain enough variability to benefit from combined augmentation methods, and that we would see an effect of combined augmentation methods with a larger test dataset.

The nnU-net modification of including sliding inference windows with Gaussian weights did improve our average metrics across test datasets. We thought that accuracy would be a better measurement for Gaussian overlap improvement rather than Edge F1-score, because the trained model penalizes misclassified edges less than misclassified axons, whereas the Gaussian overlap should increase the number of true positive and negatives regardless of the segmentation class. However, we found that the Edge F1 score improved in 9 out of the 12 models, while the accuracy only improved in 7 out of 12 models (S2 Table). A possible reason Gaussian overlap did not perform better in all scenarios is that TrailMap already ensures no voxel was located at the edge of the input when it is segmented because it segments a smaller output from a larger input.

A further area of study could be to compare the performance on models trained on specific brain areas vs. generalized models. There was a large difference in results between the moderately to highly populated cubes and the sparsely populated cube, with the fine-tuned model performing worse with the sparsely populated cubes but better with the other cubes than the original model. These results suggest that even within one brain, the variation warrants further validation in individual sections. This variation indicates the problem of complete mapping of neuronal structures into a co-registered system will likely require many structure-specific solutions in the short-term.

In addition to providing a new fine-tuned model, we also provided a roadmap to test similar fine-tuning modifications on specific structures.

## Supporting information

S1 Table Difference in metrics between model trained with 1.0 background weight, all the layers trainable, 0.001 learning rate, and rotation augmentation (Rotate); and the original model for each test dataset. Positive values indicate an improvement due to fine-tuning.
(DOCX)

S2 Table Difference in metrics using Gaussian overlap compared with inference without Gaussian for the model trained with 1.0 background weight, all the layers trainable, 0.001 learning rate, and rotation augmentation (Rotate); and the original model. Positive values indicate an improvement due to Gaussian overlap.
(DOCX)
