# Supplementary material for: Fine-tuning TrailMap: The utility of transfer learning to improve the performance of deep learning in axon segmentation of light-sheet microscopy images
Source: PLoS One. 2024 Mar 29;19(3):e0293856. doi: 10.1371/journal.pone.0293856 (PMC10980229; doi:10.1371/journal.pone.0293856)
Supplement: S1 Table — Positive values indicate an improvement due to fine-tuning. (DOCX) [file pone.0293856.s001.docx]

**S1.** Difference in metrics between model trained with 1.0 background weight, all the layers trainable, 0.001 learning rate, and rotation augmentation (Rotate); and the original model for each test dataset. Positive values indicate an improvement due to fine-tuning.

| Test Cube | Adjusted Accuracy | Axon Precision | Edge Axon Precision | Axon Recall | F1 Score | Edge F1 Score |
| --- | --- | --- | --- | --- | --- | --- |
| 1 | 0.0227 | 0.1 | 0.0632 | 0.0207 | 0.0653 | 0.0393 |
| 2 | 0.0239 | 0.0568 | 0.0736 | 0.0142 | 0.0465 | 0.0486 |
| 3 | 0.0264 | 0.0996 | 0.1807 | 0.063 | 0.0966 | 0.1312 |
| 4 | -0.0498 | -0.1037 | -0.0731 | 0.2759 | -0.0132 | 0.0565 |
| 5 | 0.0101 | 0.0192 | 0.0697 | 0.0214 | 0.0212 | 0.0469 |
| 6 | 0 | 0.1272 | 0.026 | -0.144 | -0.0627 | -0.1148 |
