# Supplementary material for: Fine-tuning TrailMap: The utility of transfer learning to improve the performance of deep learning in axon segmentation of light-sheet microscopy images
Source: PLoS One. 2024 Mar 29;19(3):e0293856. doi: 10.1371/journal.pone.0293856 (PMC10980229; doi:10.1371/journal.pone.0293856)
Supplement: S2 Table — Positive values indicate an improvement due to Gaussian overlap. (DOCX) [file pone.0293856.s002.docx]

**S2.** Difference in metrics using Gaussian overlap compared with inference without Gaussian for the model trained with 1.0 background weight, all the layers trainable, 0.001 learning rate, and rotation augmentation (Rotate); and the original model. Positive values indicate an improvement due to Gaussian overlap.

|  |  | Adjusted Accuracy | Axon Precision | Edge Axon Precision | Axon Recall | F1 Score | Edge F1 Score |
| --- | --- | --- | --- | --- | --- | --- | --- |
| Rotate | 1 | -0.0008 | -0.0076 | -0.0009 | 0.006 | -0.0013 | 0.0031 |
| Rotate | 2 | -0.0006 | -0.0018 | -0.0009 | 0.001 | -0.001 | -0.0001 |
| Rotate | 3 | -0.0005 | -0.0024 | -0.0033 | 0.0009 | -0.0015 | -0.0014 |
| Rotate | 4 | 0.0003 | 0.0005 | 0.001 | 0.0008 | 0.0007 | 0.001 |
| Rotate | 5 | 0.0015 | 0.003 | 0.0044 | -0.0018 | 0.0019 | 0.0013 |
| Rotate | 6 | 0.0002 | 0.0169 | -0.0012 | 0.003 | 0.0066 | 0.0025 |
| Original | 1 | -0.0002 | -0.0004 | -0.0016 | -0.0017 | -0.0009 | -0.0016 |
| Original | 2 | 0.0032 | 0.007 | 0.007 | 0.0006 | 0.0055 | 0.0044 |
| Original | 3 | 0.001 | 0.0033 | 0.0056 | -0.0018 | 0.0023 | 0.0028 |
| Original | 4 | -0.0002 | -0.0009 | -0.0023 | 0.0073 | 0.002 | 0.0022 |
| Original | 5 | 0.0014 | 0.0026 | 0.004 | 0.0051 | 0.0035 | 0.0045 |
| Original | 6 | 0.0005 | 0.0175 | 0.0029 | 0.0129 | 0.0149 | 0.0106 |
